# Augmented Reality for Therapeutic Education in Patients with Diabetes: Short- and Mid-Term Learning Benefits

**DOI:** 10.3390/s25041017

**Published:** 2025-02-08

**Authors:** Marcelo Calle, Francisco Abad, M.-Carmen Juan

**Affiliations:** Instituto Universitario de Automática e Informática Industrial, Universitat Politècnica de València, C/Camino de Vera, s/n, 46022 Valencia, Spain; ancalbus@upv.edu.es (M.C.); fjabad@dsic.upv.es (F.A.)

**Keywords:** augmented reality, therapeutic education, diabetes, carbohydrate choices, learning, short-term, mid-term, assessment, user experience, user performance

## Abstract

This work aims to evaluate the effectiveness of knowledge transfer through an Augmented Reality (AR) application, assessing short- and mid-term retention in children and adults with Type 1 diabetes. One objective is to determine if the AR application helps patients learn about the carbohydrate content of different foods (N = 27 Ecuadorian patients). Another objective is to evaluate the usability and satisfaction perceived by the patients. An additional objective is to compare the data from our study in Ecuador with data from a similar study conducted with Spanish children (N = 42). The results show that the AR application is effective for short-term knowledge transfer (*p* < 0.001) and has a suggestively significant effect on mid-term retention (*p* < 0.05). The AR application had an equalizing effect on knowledge outcomes between the groups (Ecuador and Spain) despite initial differences. The AR application significantly increased patients’ knowledge (*p* < 0.001) and was effective for both children and adults. Patient satisfaction was high, and learning outcomes were not influenced by age or gender. The AR application is effective for short-term knowledge transfer and mid-term retention, benefiting children and adults regardless of gender. The patients’ experience was very positive. Therefore, the AR application is a valuable tool for therapeutic education in diabetes since it offers support that is easily accessible on mobile devices, enabling autonomous learning, and it contributes to the creation of innovative, patient-centered healthcare solutions.

## 1. Introduction

Diabetes mellitus, often called diabetes, is a chronic condition characterized by the body’s inability to produce enough insulin or to use the insulin it produces effectively. Insulin, a hormone generated by the pancreas, facilitates the entry of glucose from food into the body’s cells for storage or conversion into energy. Insufficient insulin leads to excessively high blood glucose levels (hyperglycemia), which can cause severe long-term macrovascular and microvascular complications. The three main types of diabetes are Type 1, Type 2, and gestational diabetes mellitus. In Type 1 diabetes, the pancreas produces little or no insulin. This is because the body’s immune system attacks and destroys the beta cells responsible for insulin production. Type 1 diabetes primarily affects children and young adults, requiring continuous insulin therapy for survival. Type 2 diabetes develops when the body either does not produce enough insulin or becomes resistant to its effects. It primarily develops in adults, though its prevalence among adolescents is increasing. Treatment includes control of diet and exercise and oral antidiabetic medications; in advanced stages, insulin therapy may be required. Gestational diabetes mellitus happens when the body is unable to produce or properly use enough insulin during pregnancy.

According to the World Health Organization, approximately 422 million people worldwide are affected by diabetes, most of whom live in low- and middle-income countries. Diabetes is directly linked to 1.5 million deaths annually. According to the International Diabetes Federation (https://diabetesatlas.org/data/en/ (accessed on 10 December 2024)), the global prevalence of diabetes is projected to increase to 643 million by 2030 and 783 million by 2045.

Therapeutic education in diabetes (TED) refers to a structured process of teaching people with diabetes about the nature of their condition, how to manage it, and how to integrate effective self-care practices into their daily lives. This education includes understanding diabetes, blood glucose monitoring, insulin administration, nutrition and diet, physical activity, prevention of complications, psychosocial support, and self-management skills. TED encourages patients to take responsibility for their disease management.

Proper nutritional management is critical in preventing insulin resistance, slowing its progression, and avoiding neurological and vascular complications resulting from poor glycemic control. Therefore, nutrition is a key component of educational programs for all diabetes patients. Nutritional guidelines emphasize regulating daily caloric intake and its distribution across meals, as well as balancing fats, proteins, and carbohydrates. Because carbohydrates directly affect blood glucose levels, understanding the appropriate carbohydrate intake for each meal is essential for effective disease management. This regulation helps achieve a balanced diet and maintain near-normal blood glucose levels.

The integration of assistive technology systems can enhance TED. Extended reality (encompassing Virtual Reality (VR), Augmented Reality (AR), and Mixed Reality (MR)) offers promising avenues to enhance TED. Notably, AR has been widely employed in educational applications (e.g., [1,2]), where virtual elements are superimposed onto real-world images [3] to enhance the learning experience. Previous works have demonstrated that AR can improve learning outcomes [1]. In addition, numerous review publications, both general [1,2] and specific, have highlighted the applications of AR in areas such as language learning [4], mathematics [5], chemistry [6], natural sciences for preschool and primary education [7], education for students with educational needs [8], support for individuals who are deaf or hard of hearing [9], and even in the military training contexts [10].

The motivation for this research arises from the growing need for innovative solutions to enhance TED for patients. Traditional educational methods often fail to engage patients effectively. Developing autonomous, easily accessible, and user-friendly tools would enable patients to access these resources more conveniently. The contributions of this work include the adaptation of an AR application tailored for diabetes patients in Ecuador, offering an engaging and accessible approach to therapeutic education in diabetes. The study sample comprises both children and adults. Short- and mid-term learning outcomes are assessed, and the results of learning through the AR application are compared with traditional educational methods. Furthermore, data from Ecuadorian patients are compared with those from Spanish patients. In summary, this work demonstrates the potential of AR-based tools to provide a more engaging complement to conventional methods and highlights their applicability across diverse cultural and demographic contexts. The findings aim to pave the way for broader implementation of similar tools, addressing the global challenge of improving chronic disease management through innovative, patient-centered approaches.

## 2. Related Work

### 2.1. Virtual Reality

VR is a technology with significant potential to assist diabetes patients, attracting considerable interest from researchers, as highlighted by recent reviews [11,12]. Hao et al. [11] conducted a systematic review to identify, critically evaluate, and synthesize the effects of VR on balance in individuals with diabetes. They concluded that VR-based rehabilitation shows promising effects in enhancing balance among individuals with diabetes. Vaughan [12] offers a comprehensive review of VR and AR applications in diabetes, highlighting key areas of use and providing a foundation for future developments in VR tools for diabetes management. He concluded that VR and AR in diabetes show potential to improve the training of diabetologists while advancing education, prevention, and treatment strategies for both adults and children with Type 1 or Type 2 diabetes. This section explores the use of VR as a tool for managing and treating diabetes, focusing on its applications in education, prevention, and patient care for individuals with Type 1 and Type 2 diabetes.

For individuals with Type 1 diabetes, different VR tools have been presented. For instance, a platform incorporating a variety of scenarios was created to train individuals with diabetes [12]. These include training for exercising safely in a VR gym while preventing hypoglycemia for individuals with Type 1 diabetes, advice on carbohydrate counting in a VR kitchen, training on finger-prick techniques for newly diagnosed patients, and a workplace scenario for glucose monitoring. The platform was tested on Android smartphones using Google Cardboard. Similarly, a virtual world game was created to support children with Type 1 Diabetes, suggesting that virtual games should incorporate three essential elements: (1) The player’s connection with the game’s protagonist; (2) a reward system for adhering to medication and diet; and (3) a focus on the disease’s biochemical basics [13]. Lanning et al. [14] created a four-part VR intervention to expose adults with Type 1 diabetes to expected Closed Loop system barriers: perceived hassles of using Closed Loop, body image, unwanted social attention, and deskilling fears. The authors concluded that the VR intervention showed great potential for addressing expected barriers in the uptake and use of Closed Loop systems while maintaining enthusiasm and preserving positive expectations toward Closed Loop.

For individuals with Type 2 diabetes, VR tools have also been developed. For instance, Diabetes Island was developed within a virtual world in Second Life [15]. This virtual island provides a range of diabetes self-care education activities and resources. The users, identified by their avatar name, could communicate through voice and text chat tools. On Diabetes Island, the participants accessed various educational resources, including scenarios on nutrition labels, educational signage, and a learning center with written materials and videos. Four interactive scenarios focused on healthy eating and physical activity. Health professionals, as avatars, led real-time formal and informal sessions on topics like healthy eating, medication adherence, physical activity, and diabetes care. The participants earned points for engaging in activities, which could be traded for avatar clothing. The study focused on African American women with Type 2 diabetes, characterized by low levels of physical activity and high-fat diets. The impact of the intervention was evaluated using a single-group repeated measures design, with assessments at three time points: (1) baseline; (2) three months (mid-intervention); and (3) six months (immediately post-intervention). The Diabetes Island intervention showed (1) good participant acceptance and regular usage; (2) a reduction in diabetes-related distress, body mass index, and environmental barriers to self-care; and (3) improvements in dietary habits and physical activity. All of this was observed among a group of low-income patients. Nintendo Wii Fit Plus (Nintendo Co., Ltd., Kyoto, Japan) has been used to develop educational games to promote physical activity and improve health outcomes in adults with diabetes. A study involving adults with Type 2 diabetes used a Wii Fit Plus game for 12 weeks [16]. The conclusions were that the Wii Fit Plus interactive exercise game effectively motivated patients to enhance physical activity, improve glucometabolic control, and boost their quality of life. Reducing diabetes-related distress is another application of VR. With this objective in mind, Ghosal et al. [17] proposed the development of a VR-based mindfulness application to reduce diabetes distress for patients with Type 2 Diabetes. On the therapeutic side, Lee et al. [18] investigated the impact of a VR exercise program (VREP) on patients with Type 2 diabetes.

The program used IoT sensors attached to an indoor bicycle connected to a smartphone, allowing the participants to engage in immersive VR exercises via a headset. The study, conducted three times weekly over two weeks, found significant reductions in mean blood glucose and serum fructosamine levels in the VREP and indoor bicycle exercise (IBE) groups compared to the control group. Although body mass index showed no significant changes, muscle mass notably increased in the VREP and IBE groups. The effects of various feedback modalities on balance within immersive VR have also been explored. For example, Mahmud et al. [19] investigated the effects of various feedback modalities (auditory, vibrotactile, and visual) on balance within immersive VR. The study included participants with balance impairments due to Type 2 diabetes as well as those without balance impairments. During standing reach-and-grasp tasks, auditory and vibrotactile feedback significantly enhanced balance for all of the participants, while visual feedback showed significant improvement only for those with balance impairments. Cinematic VR has also been explored. For example, Beverly et al. [20] carried out a pilot study to assess the effectiveness of a cinematic VR training program aimed at care providers. The program centered on an elderly patient with Type 2 diabetes and multiple geriatric syndromes, highlighting the risk of elder abuse and neglect.

### 2.2. Augmented Reality

While AR has shown promise in healthcare, its applications in TED remain relatively underexplored. Previous research works on AR that are not directly related to our work are as follows: The first one is an AR application for learning about antihypertensives for people with Type 2 diabetes [21]. The AR application scans pre-defined patterns (e.g., a medication package) and shows the augmented information. The second one is Jerry the Bear^®^, a teddy bear toy with image targets embedded in his body [22] to provide TED to children with Type 1 diabetes. For example, the AR application on a mobile device recognizes the areas of the teddy bear’s body where image targets are located. It allows the blood glucose to be measured by simulating a prick in the bear’s hand or administering insulin with a pen or pump in Jerry’s belly. The process of learning to count carbohydrates is not carried out with AR. The third one was presented by Kurniawan et al. [23], who developed a mobile AR application to provide diabetes drug information using QR codes to display 3D representations. The study, involving pharmacy students, found that AR technology supported 76.2% of student learning, demonstrating its effectiveness in enhancing educational outcomes. The fourth was presented by Fajriyah et al. [24], who conducted a study to analyze the effect of AR-based therapeutic patient education on Health Locus of Control in Type 2 diabetes patients. The AR application utilized barcode markers to trigger interactive content, focusing on the five key areas of diabetes management: education, diet, physical activity, blood glucose monitoring, and medication. It also featured quizzes to assess patient knowledge and provided explanations on managing diabetes. The intervention was administered three times a week over a period of three months. The participants were split into two groups: half in the intervention group and half in the control group. Their findings indicated that AR-based Therapeutic Education significantly improved Health Locus of Control scores in Type 2 Diabetes patients. However, no significant difference was found between the intervention and control groups in terms of overall Health Locus of Control improvement.

Previous research works that are directly related to our work are the following. The first one was presented by Domhardt et al. [25], who developed an AR application for mobile devices that estimates carbohydrate choices in real food. The application superimposes a virtual mesh over those foods. The users can interact with this virtual mesh to adjust it to the volume of the real food. The application measures the volume of real food and estimates its weight. For the correct functioning of the application, a marker must be placed in front of the food to be measured. Eight patients took part in the study. In 44% of the assessments, the margin of error decreased by a minimum of 6 g of carbohydrates. The second one is from one of our previous works [26], in which we developed the first version of the AR application for Spanish children. In the study, two post-knowledge questionnaires were used: one containing the same foods as the pre-knowledge questionnaire and a second one containing different foods from those in the pre-knowledge questionnaire. There were no statistically significant differences between the results of these two different post-knowledge questionnaires. In this work, the AR application has been adapted to food for patients from Ecuador; the sample includes children and adults. Short- and mid-term memories were evaluated. The AR outcomes were compared with traditional ones. We have also compared the Spanish data [26] with our study conducted in Ecuador.

## 3. Materials and Methods

### 3.1. Augmented Reality Application

**Figure 1:**
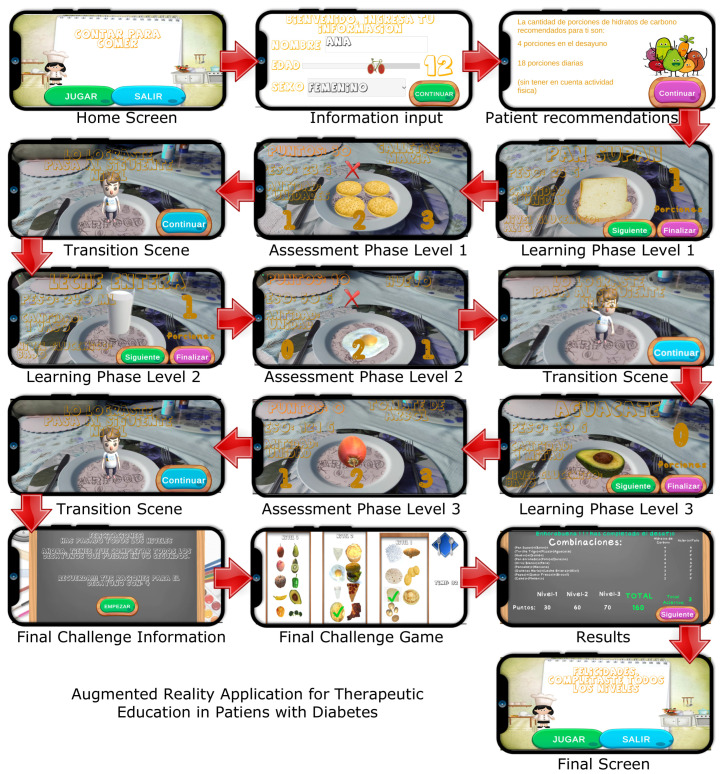
A graphical summary of the steps to be followed for the correct use of the AR application.

**Figure 2:**
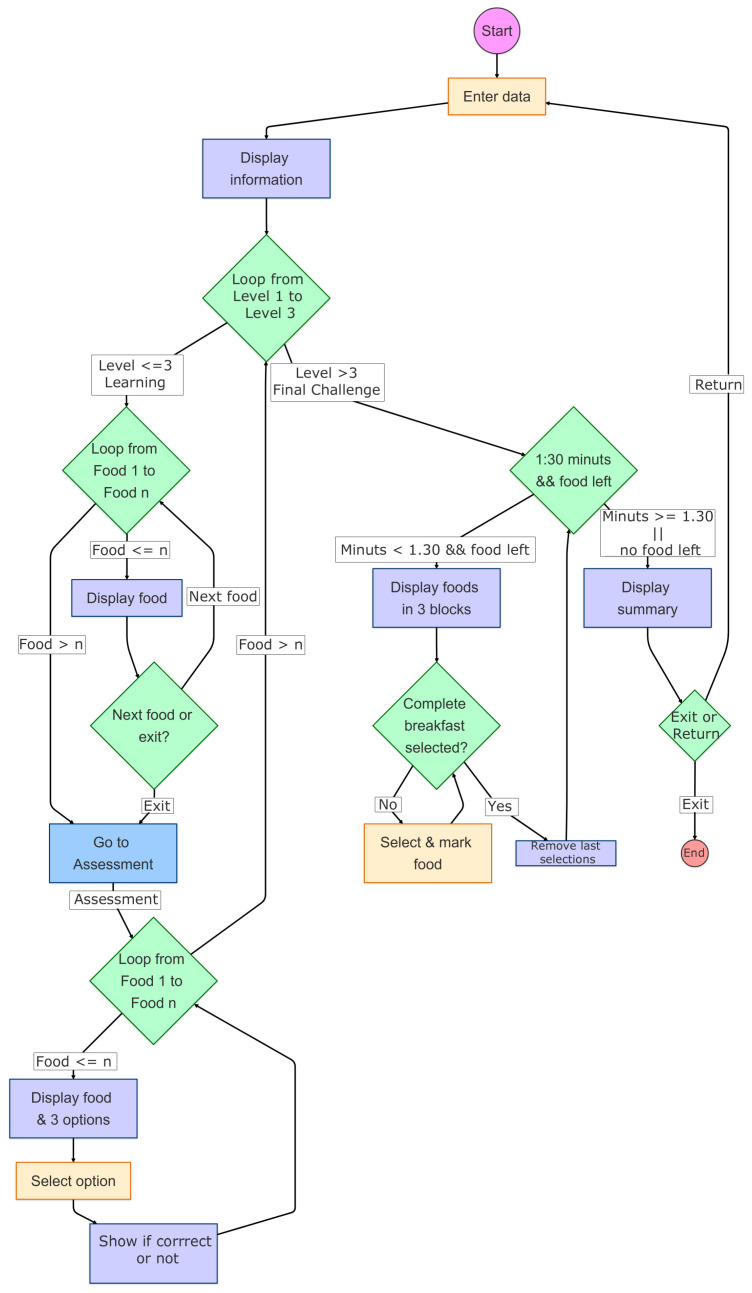
A flowchart detailing the functionality of the AR application.

The AR application shows virtual food on a real dish using a mobile device. The goal is to make users believe that they are real foods. When the application recognizes the image in the center of the dish, the virtual food is shown in the center of the physical dish. The users can place the dish wherever they like and view the food in the desired location. Users can freely move their mobile device or dish to view the virtual food from any angle (360°). The users can zoom in or out to view the food closer or farther away. The users can use the application at home to complete the training recommended by their therapists. To do so, they simply need to install the application on their mobile phone and print the image to attach to the dish they wish to use. Figure 1 provides a graphical summary of the steps that patients must follow in the AR application, while Figure 2 presents a flowchart illustrating its functionality in detail.

(1) Filling out personal information: Initially, the patients must enter their age and gender. Following this, the application shows the recommended carbohydrate choices for their age for the whole day and the carbohydrate choices for breakfast (1 carbohydrate choice = 15 g of carbohydrates). This equivalency is currently used in Ecuador, the United States, Mexico, and most of Latin America. However, it varies in other countries. For example, in Spain, one carbohydrate choice equals 10 g of carbohydrates, and in Austria, one carbohydrate choice equals 12 g of carbohydrates.
(2) The AR application: The application has three levels, each of which focuses on a distinct food category. Specifically, these categories are dairy products, grains, and fruits. The first level (dairy products) includes seven different foods, while the second level (grains) includes eight foods, and the third level (fruits) includes 10 foods. Each level begins with a learning phase where foods are shown in real size along with their weight, carbohydrate choices, glycemic level, and homemade portions of food, such as a tablespoon or cup (this is the additional information that the Foundation shows in its educational materials). After this initial learning phase, the patients undergo an assessment phase to test their knowledge. During this phase, the patients are shown each food in the center of the screen and must select the correct number of carbohydrate choices from the three options provided.
(3) The final challenge: After completing the three levels, the patients face a final challenge, which is to identify as many unique breakfast combinations as possible within a set time (one and a half minutes). Each breakfast must adhere to the recommended carbohydrate choices for the users’ ages. To succeed, the patients must select foods from at least two groups. Each breakfast entry is completed by pressing the button in the upper right corner (represented by a fork and spoon).

The AR application was developed using Unity (https://unity.com (accessed on 10 December 2024)) as the primary development platform, with Vuforia Engine (https://developer.vuforia.com (accessed on 10 December 2024)) integrated for its AR tracking and registration capabilities. Vuforia Engine provides tracking capabilities for a selection of items and areas, which can be grouped into Images, Objects, and Environments. Specifically, for the development of our AR application, we used an image target, which is glued to a real dish to make it look like it is a design of the dish itself. Another option would have been to select a dish with a design that is suitable for recognition with Vuforia Engine. This image target is recognized and tracked by the Vuforia Engine in real time using the image captured by the device’s camera. This is achieved by the Image Target Observer, which begins tracking the image target upon detection. The image is tracked by comparing the natural features extracted from the camera image with a known target stored in the active database. Once the image target is detected, the Vuforia Engine calculates its position and orientation in 3D space. If Extended Tracking is active, the pose information of an image target remains available even when it is no longer visible in the camera’s field of view, is occluded, or cannot be tracked for other reasons. Extended Tracking uses the device’s pose to improve tracking performance and ensure continuity of tracking, even when the target is out of view. In practice, Extended Tracking means that once the device is moved away from the original target, the augmentations stay in place relative to the real world and remain consistent with the reference frame defined by the target. The Device Pose Observer obtains tracking information about the device’s position and orientation in the physical world by analyzing the visual features of the environment captured by the camera and, in certain cases such as Extended Tracking, by using the built-in inertial measurement unit (IMU) sensor to determine the device’s six-degree-of-freedom pose. Typical sensors in a mobile device IMU include an accelerometer, gyroscope, and magnetometer. For virtual content mapping, Vuforia Engine anchors the virtual food to the image target’s position and dynamically adjusts the rendering to maintain proper alignment as the user or the device moves. The virtual elements (food) were adjusted in Unity on the image target so that when displayed on the screen, they are shown in real size according to their carbohydrate portions. The physical dish, as a movable, rotatable, and inclinable object, serves as a Tangible User Interface by enabling intuitive interaction with the digital representation of food in AR, seamlessly bridging the physical and digital worlds. The virtual guide is displayed using the center of the image target as the origin of the coordinate system to show the other information, providing users with step-by-step instructions or contextual information. Vuforia Engine supports different operating systems, tools, and device versions for developing and running AR applications (https://developer.vuforia.com/library/platform-support/supported-versions (accessed on 10 December 2024)). Our AR application can run on mobile devices with an Android operating system and an iOS operating system. In our study, we used a Xiaomi POCO X3 NFC smartphone (Xiaomi Corporation, Beijing, China).

### 3.2. Participants

The study participants are patients who attended one of the activities organized by the Diabetes House, which is based in the cities of Cuenca and Portoviejo (Ecuador). The Diabetes House (https://casadeladiabetes.org.ec (accessed on 10 December 2024)) has therapeutic learning activities as well as educational material that can be accessed from its website for patients with Type 1 diabetes. A total of 27 patients from Ecuador with Type 1 diabetes (14 men and 13 women), ranging in age from 5 to 28 years old, participated in the study. The mean age was 15.85 ± 5.92 years old. Information about the study was provided to the patients or their parents (in cases where the patients were minors). Informed consent forms were signed by the patients or their parents before participating. The study adhered to the principles of the Declaration of Helsinki and received approval from the Ethics Committee of the University of Cuenca (Ecuador).

### 3.3. Measures

Assessment of the knowledge: To assess the knowledge about carbohydrate choices, there was a knowledge questionnaire in which the patients should indicate the number of carbohydrate choices for nine foods with images selected from the 25 foods shown in the application. All of the foods that appeared on the questionnaire were included in our AR application and the traditional session, and the patients were informed of their carbohydrate choices. The same questionnaire was used to measure the level of knowledge of carbohydrate choices at different stages of the study. The knowledge variable is the sum of correct answers (from 0 to 9) and can be expressed mathematically as the following:

Knowledge Score = Σ Correct Answers, where each correct answer is assigned a value of 1, and each incorrect answer a value of 0.

The following questionnaires were used:

(1) Short-UEQ (User Experience Questionnaire) [27]: The UEQ consists of eight items on a 7-point Likert scale, grouped into three variables: Pragmatic Quality, Hedonic Quality, and Overall. We used the short questionnaire instead of the long one with 26 items so that the session would not be too tedious.
(2) Questions: Thirteen questions were included to assess usability and satisfaction with the application. These questions were the same as those used in [26].
(3) Data: This questionnaire gathered data from the participants, including their age and gender.

### 3.4. Protocol

The initial study design was a between-subjects design. This implied two separate groups: a control group using traditional learning and an experimental group using the AR application for learning. The study began with the control group. The knowledge outcomes were analyzed, and it was observed that the patients had not significantly increased their knowledge about carbohydrate choices. As a result, it was decided to continue with this group of patients and use a within-subjects design. The objective was to test whether the same patients could learn significantly using the AR application. Therefore, the protocol used in the study presented in this manuscript involved three sessions.

Session 1: The patients learn by attending a traditional class. The steps followed by the patients were as follows:

- Fill out the knowledge questionnaire;
- Attend a traditional session;
- Fill out the knowledge questionnaire.

Session 2: The patients learn by using the AR application. The session took place one week after Session 1. The steps followed by the patients were as follows:

- Fill out the knowledge questionnaire;
- Learn by using the AR application;
- Fill out the knowledge questionnaire;
- Fill out the rest of the questionnaires (usability, satisfaction, and data questionnaires).

Session 3: The patients fill out the knowledge questionnaire to test their recall of what has been learned. The session took place two weeks after Session 2. This test focused on the effects of learning on mid-term memory.

A nutritionist taught the traditional class as she usually does in her regular classes. The session lasted one hour. In the class, images of the food were shown on a real scale and cut out in the shape of the food. The nutritionist talked about the foods included in the AR application and presented all of the data shown in the AR application. All of the foods included in the test were included in the class. This class also explained other concepts, such as the healthy plate. For example, she explained how these foods can be combined to make a healthy meal. A round of questions was also included after the explanation of each food. Some of them were: Can I eat my healthy dish in parts? Is it advisable to eat five meals a day? How can I determine the food choices that are not in the guide? The patients also asked for recommendations on preparing food (homemade recipes and deviating from the guide). The nutritionist answered all of the questions.

The study was conducted at the Diabetes House facilities in Cuenca and Portoviejo (Ecuador) in one session on a Saturday morning (from 10:00 a.m. to 12:00 a.m.). The patients required approximately 30 min to use the AR application and complete the questionnaires.

## 4. Results

The Shapiro–Wilk test was employed to assess the normal distribution of the variables. Most variables exhibited a non-normal distribution. Non-parametric tests were used for the entire dataset, as they are more appropriate for such distributions. The statistical data are reported as (*Mdn*: median; *IQR*: interquartile range), and all test results are provided as (test *U*: Mann–Whitney U/test *W*: Wilcoxon Signed-rank, normal approximation *Z*, *p*-value, *r* effect size). Results with *p* < 0.005 are considered to be statistically significant and are highlighted in bold with the symbol ‘*’. Results with 0.005 ≤ *p* < 0.05 are considered to be suggestively significant and are highlighted in bold [28]. Statistical analysis of the data was conducted using the R (4.1.3) statistical toolkit (http://www.r-project.org (accessed on 10 December 2024)).

### 4.1. Short-Term Learning Outcomes

Assessing short-term learning outcomes is crucial for understanding the immediate impact of the educational method. To evaluate this, the Wilcoxon Signed-rank test was used to test whether the patients learned using the traditional method (*W* = 43.5, *Z* = −1.178, *p* = 0.355, *r* = 0.160). This was performed by comparing the scores obtained before (*Mdn* = 4.0; *IQR* = 2.0) and after the traditional learning session (*Mdn* = 4.0; *IQR* = 2.0).

As mentioned, since no statistical differences were found between the pre-test and the post-test, the same patients used the AR application one week later. The Wilcoxon Signed-rank test was used to test whether the students learned using the AR application (*W* = 0.0, *Z* = −4.526, *p* < 0.001 *, *r* = 0.616). This was performed by comparing the scores obtained before (*Mdn* = 4.0; *IQR* = 3.0) and after (*Mdn* = 8.0; *IQR* = 1.0) using the AR application.

**Figure 3:**
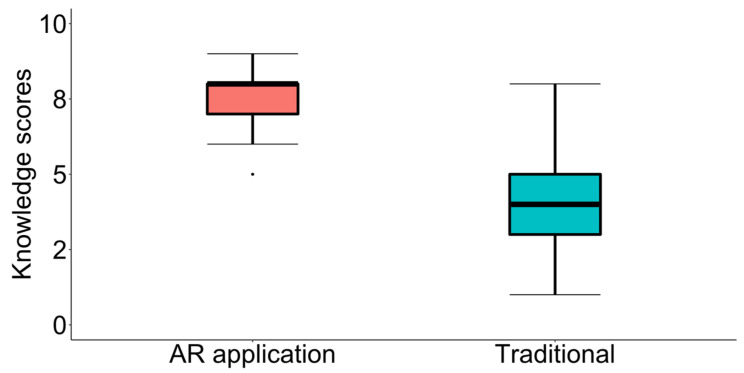
Box plot for the scores obtained by the patients after learning to use the AR application and the traditional method.

The Wilcoxon signed-rank test was used to check whether there were differences in the scores of the same patients when using the two learning methods (*W* = 0.0, *Z* = −4.546, *p* < 0.001 *, *r* = 0.619). This was performed by comparing the score obtained using the traditional method (*Mdn* = 4.0, *IQR* = 2.0) and the score obtained using the AR application (*Mdn* = 8.0, *IQR* = 1.0). Figure 3 shows a box plot for the scores obtained by the patients after learning using the AR application and the traditional method.

From these results, we can conclude that using the AR application for short-term knowledge transfer was effective and offered a statistically significant improvement over the traditional method.

To determine the learning performance, we compared the knowledge outcomes of our study with those of a similar study of Spanish children [26]. The same experimental conditions were recreated in both studies. The main difference between the two AR applications was the food used, which in both cases was adapted to the country in which the studies were conducted. The steps in the studies related to the validation of the AR application were similar. The objective of this comparison was to determine whether the application was effective in both countries, taking into account their cultural, educational, social, and healthcare system access disparities. These factors may influence the effectiveness of the AR application in both countries. The Spanish study [26] compared the use of the same and different questionnaires for the pre-test and the post-test. In our study, the group of 42 children that used the same questionnaire as pre-test and post-test is used for comparison. The children ranged in age from five to fourteen years old. The mean age was 8.74 ± 2.56 years old. There were 24 boys (57.1%) and 18 girls (42.9%).

The initial knowledge assessment establishes a baseline that accounts for potential differences related to the time elapsed between the two studies, as well as inherent differences between the two populations (Spanish and Ecuadorian) and other factors, such as cultural, educational, social, and healthcare system access disparities.

Mann–Whitney U tests were applied to test whether there were differences between the initial knowledge of the Ecuadorian and Spanish children and their knowledge after using the AR applications. When comparing the initial knowledge of the group of children from Ecuador (*Mdn* = 4.0; *IQR* = 3.0) with the group of children from Spain (*Mdn* = 2.0; *IQR* = 3.0), the results were as follows: *U* = 870, *Z* = 3.782, *p* < 0.001 *, *r* = 0.455. This difference in favor of the Ecuadorian patients can not only be attributed to advances in educational resources and technology but also to differences in the health education strategies applied in each country. When comparing the knowledge after using the AR applications of the group of children from Ecuador (*Mdn* = 8.0; *IQR* = 1.0) with the group of children from Spain (*Mdn* = 7.0; *IQR* = 3.0), the results were as follows: *U* = 577.5, *Z* = 0.133, *p* = 0.899, *r* = 0.016. The small difference in favor of the Ecuadorian children can be explained by the significant difference in initial knowledge. In any case, although the initial knowledge of the two groups was different, the knowledge demonstrated after using the AR applications was not significantly different statistically. These results also suggest that the AR applications significantly increased patients’ knowledge to high levels, with many scores at or near the maximum.

Since the ages of the two groups (Spaniards and Ecuadorians) differ, a subgroup of Ecuadorians was selected to include only participants aged 14 or younger. Mann–Whitney U tests were applied to test whether there were differences between the initial knowledge of the Ecuadorian subgroup and the Spanish children, as well as differences in their knowledge after using the AR applications. Comparing the initial knowledge of the group of children from Ecuador (*Mdn* = 5.0; *IQR* = 2.25) with the group of children from Spain (*Mdn* = 2.0; *IQR* = 3.0), the results were as follows: *U* = 429.5, *Z* = 3.784, *p* < 0.001 *, *r* = 0.515. When comparing the knowledge after using the AR applications of the subgroup of children from Ecuador (*Mdn* = 8.0; *IQR* = 1.0) with the group of children from Spain (*Mdn* = 7.0; *IQR* = 3.0), the results were as follows: *U* = 260.5, *Z* = 0.183, *p* = 0.863, *r* = 0.025. Therefore, after removing participants older than 14 years old from the Ecuadorian group, the results remain consistent with the previous ones: statistically significant differences in initial knowledge were observed between the groups, but no differences were found in final knowledge after using the AR applications.

Mann–Whitney U tests were applied to test whether there were differences between the knowledge of the groups of Ecuadorian adults (aged 18 or older) and Ecuadorian children after using the AR applications. Comparing the knowledge demonstrated after using the AR application of the Ecuadorian adult group (*Mdn* = 8.0; *IQR* = 0.75) with the Ecuadorian children group (*Mdn* = 8.0; *IQR* = 1.0), the results were as follows: *U* = 85.0, *Z* = 0.0, *p* = 1.0, *r* = 0.0. Mann–Whitney U tests were applied to test whether there were differences between the knowledge of the groups of Ecuadorian adults (aged 18 or older) and Spanish children after using the AR applications. Comparing the knowledge demonstrated after using the AR applications of the Ecuadorian adult group (*Mdn* = 8.0; *IQR* = 0.75) with the Spanish children group (*Mdn* = 7.0; *IQR* = 3.0), the results were as follows: *U* = 215.5, *Z* = 0.132, *p* = 0.905, *r* = 0.018. These results suggest that AR applications are effective for both children and adults.

### 4.2. Mid-Term Learning Outcomes

Assessing mid-term learning outcomes is crucial for understanding the method’s ability to promote sustained knowledge retention over time. To evaluate this, only the patients who completed all of the learning questionnaires were included in the analyses of this section. The pre-session test with the AR application was used to verify the patient’s retention of what was learned in the traditional learning session. The Wilcoxon Signed-rank test was used to check this retention (*W* = 19.5, *Z* = 1.769, *p* = 0.071, *r* = 0.347) with the post-test of the traditional session (*Mdn* = 4.0; *IQR* = 3.0) when compared with the pre-test of the AR session (*Mdn* = 4.0; *IQR* = 3.0). The Wilcoxon Signed-rank test was also used to check the difference between the initial knowledge (*Mdn* = 4.0; *IQR* = 3.0) before using the traditional method and one week after the learning session (*Mdn* = 4.0; *IQR* = 3.0), with the results: *W* = 8, *Z* = 1.041, *p* = 0.345, *r* = 0.204. The results demonstrate that there were no statistically significant differences between the two analyses. While the patients exhibited slight learning in the traditional session, this learning was not maintained after one week, resulting in a return to a level of knowledge similar to that observed at the beginning.

The test performed two weeks after the learning session with the AR application was used to verify the patients’ retention of what was learned in that session. The Wilcoxon signed-rank test was used to test this retention (*W* = 65, *Z* = 2.887, *p* < 0.005 *, *r* = 0.566), comparing the post-session test using the AR application (*Mdn* = 8.0, *IQR* = 1.0) with the two-week post-session test (*Mdn* = 6.0, *IQR* = 2.0). The Wilcoxon signed-rank test was also used to test the difference between the initial knowledge before the learning session with the AR application (*Mdn* = 4.0; *IQR* = 3.0) and two weeks after the learning session (*Mdn* = 6.0; *IQR* = 2.0), with the results: *W* = 10, *Z* = −1.974, ***p* = 0.044**, *r* = 0.387. The results demonstrate that there were significant differences in both analyses. The results of the first analysis indicate that the patients had statistically significant less knowledge than they did two weeks earlier. The results of the second analysis indicate that the patients had suggestively significant more knowledge than they had before the learning session with the AR application. Therefore, the patients retained a significant amount (but not all) of what they learned using the AR application in the mid-term. From all of these analyses, it can be concluded that the use of the AR application did indeed have a suggestively significant effect on mid-term knowledge retention.

### 4.3. User Experience

**Figure 4:**
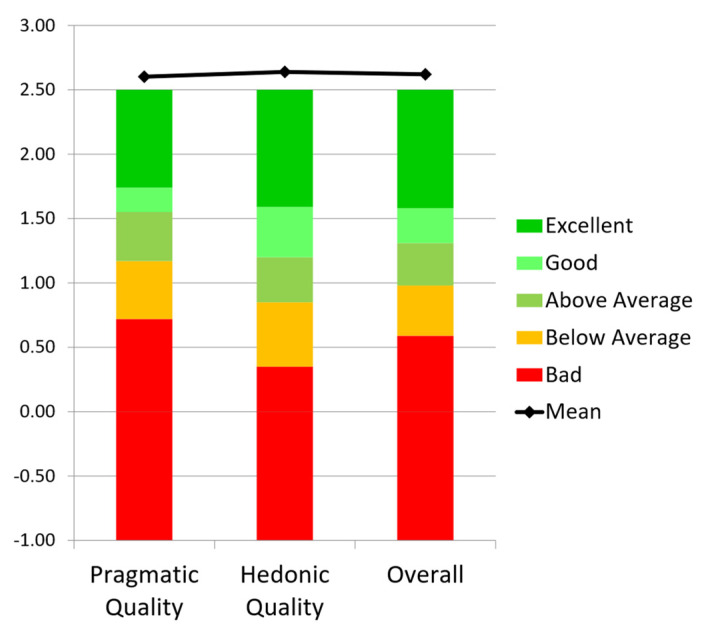
Comparison of the patient’s responses after using the AR application with the benchmark provided in the UEQ Data Analysis Tool.

The patient’s responses to the short-UEQ questionnaire after using the AR application were analyzed using the UEQ Data Analysis Tool (https://www.ueq-online.org/ (accessed on 10 December 2024)). By using one of the UEQ downloadable Excel sheets, a graph was created (Figure 4) to evaluate the quality of our AR application in comparison to the products in the benchmark dataset included in the UEQ Data Analysis Tool [29]. This graph shows that the AR application performs exceptionally well relative to the UEQ benchmark. Based on the interpretation guidelines provided in the UEQ tool and in [29], our AR application ranks within the top 10% for all three variables or scales.

Table 1 shows that 7 of the 13 comparisons show significant differences in favor of the Ecuadorians (three of them are statistically significant, and four of them suggestively significant). However, the scores are high enough to indicate that both groups are highly satisfied with the AR applications.

### 4.4. Gender and Age

**Figure 5:**
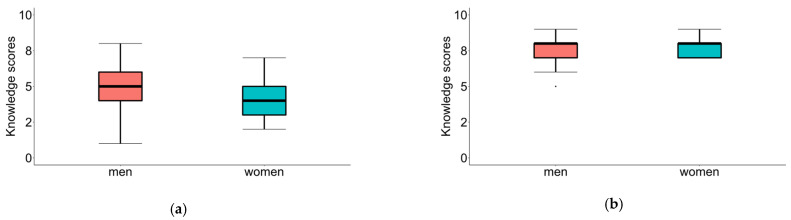
Box plots for the scores obtained by men and women after learning using (**a**) the traditional method and (**b**) the AR application.

To determine if gender influences the outcomes obtained by the patients, we applied the Mann–Whitney U tests. The learning outcomes of women and men were compared after using the traditional method and after using the AR application. The results are shown in Table 2. No significant differences were found for either of the two analyses. Figure 5 shows box plots for the scores obtained by women and men after learning using the traditional method and the AR application.

We used the Mann–Whitney U tests to assess whether gender affects the UEQ variables. The UEQ variables for women and men were compared. Table 3 shows the results. None of the analyses revealed significant differences. We used the Mann–Whitney U tests to assess whether gender affects the satisfaction and usability variables. These variables for women and men were compared. None of the analyses revealed significant differences and are not included in this paper.

The Kruskal–Wallis tests were applied to assess whether age has an impact on any of the learning questionnaires filled out by the patients (Table 4). Kruskal–Wallis tests were also used to determine whether age affected the UEQ variables (Table 5) and the usability and satisfaction questionnaire variables. None of the analyses revealed significant differences.

## 5. Discussion

In this work, we present a mobile AR application to support TED and to help patients learn to count carbohydrates. Our AR application recognizes a real dish and shows virtual food on it, giving the patient the feeling that both are real. The food was adapted for patients from Ecuador. In general, managing one’s diet is crucial for maintaining good health. Monitoring their food intake is essential [30] for patients with diabetes. The quantity of carbohydrates consumed during meals directly affects the insulin dosage required. Therefore, knowing the amount of carbohydrates in foods would empower patients to manage their condition independently and enhance their glycemic control. Carbohydrate counting is often challenging for patients with Type 1 diabetes, and inaccurate counting is the primary identified cause of poor postprandial glycemic control [31]. The nutrition guidelines for patients with Type 1 diabetes recommend teaching them how to count carbohydrates using tools tailored to their preferences [31]. Our AR application could serve as one of these tools, providing an interactive and personalized approach to support patients in carbohydrate counting. Our study did not examine enhancements in patients’ glycemic control using our app. Nonetheless, this remains a significant area for future research.

Our application runs on mobile devices. Considering that the global smartphone penetration rate was estimated at 69% in 2023, a mobile application is an optimal option for reaching the widest audience possible. In our study, we monitor the learning process. Still, the application can be used independently at any time and any place, enhancing flexibility in learning as highlighted in other research [32]. Additionally, previous works also suggest that flexible and technological approaches could be used to reach young audiences [33]. Such applications can be particularly beneficial in rural areas, where challenges such as lower health literacy and limited access to diabetologists often hinder effective diabetes management [20].

In terms of learning about carbohydrate choices through our AR application in the short-term, the statistically significant differences in patients’ knowledge before and after using the application demonstrate that such tools are effective for knowledge transfer. This result aligns with other studies on AR, which have shown that using mobile AR for educational purposes can enhance the learning process [1,32]. The AR application is also effective for both children and adults, regardless of gender.

In terms of learning about carbohydrate choices through our AR application in the mid-term, the patients retained a suggestively significant amount (but not all) of what they learned using the AR application in the mid-term. Similarly, Alhamad & Agha [34] conducted a study comparing knowledge acquisition and retention between mobile learning and traditional learning in teaching respiratory therapy to third-year students. The lesson focused on arterial blood gases. Knowledge assessments were conducted before the class, immediately after, and again two weeks later. Their findings on mobile learning align with those of our study on the AR application, indicating that students gained knowledge after training and retained it over time, though retention declined compared to the immediate post-training assessment. Another study that also assessed knowledge retention two weeks after a learning session was conducted by Gargrish et al. [35]. They assessed memory retention among K-12 students using AR for geometry. The first evaluation took place immediately after the learning session, the second was conducted two weeks later, and the third occurred after two months. Their results showed that the highest scores were obtained immediately after learning, followed by a decline in retention at the two-week mark, with a slight further decrease at the two-month mark. Their findings regarding the initial knowledge gain and the decline in knowledge retention at the two-week mark align with our study’s results.

Using the traditional method, the patients did not significantly improve their initial knowledge. Our explanation for this result is that the length of the session, which was one hour and included more information beyond just carbohydrate choices, potentially overwhelmed the patients, hindering their ability to remember specific data such as the carbohydrate choices of the foods used in the test. However, it is important to acknowledge that traditional teaching methods are effective and have been successfully used for many years. Moreover, other works have compared the learning outcomes using AR applications and traditional methods and found no statistically significant differences [32,36]. Therefore, a new study should be conducted to determine the optimal type and duration of traditional sessions needed to achieve knowledge equivalent to an AR session of 30 min.

Chiang et al. [37] compared a group using a mobile AR application and a group using a mobile application without AR. Their findings indicated that the average learning outcomes were significantly higher in the AR group compared to the non-AR group. This demonstrates the potential of AR in enhancing educational outcomes, which is in line with the results obtained in our work.

In terms of usability, several researchers have highlighted its significant impact on educational effectiveness [38]. According to Sun et al. [39], systems that are user-friendly enable learners to concentrate more on the content. In our study, our application was highly intuitive (a median of 5 on a scale from 1 to 5 in US#1), and the patients rated their user experience on the Short-UEQ questionnaire as excellent, scoring in the top 10% on all three variables relative to the UEQ benchmark [29]. Therefore, we can argue that our application helps patients focus on learning about the carbohydrate content of different foods.

The patients expressed a strong interest in using these applications to expand their knowledge about diabetes, scoring a median of 5 with an interquartile range of 0 (on a scale of 1 to 5). This suggests that the application would be widely welcomed as a valuable educational tool in therapy. We consider our application to be a valuable educational resource due to its tailored approach to the needs of diabetes patients and its strong motivational impact in presenting or reinforcing information about carbohydrate choices.

Finally, the current state of AR, MR, and robotics, as well as their expected evolution, suggests that the range of possibilities for developing new tools for therapeutic education in diabetes is vast. With headsets such as Apple Vision Pro offering advanced capabilities, the potential for creating immersive, interactive, and effective therapeutic education tools has never been greater.

## 6. Conclusions

This work introduces a mobile AR application that is designed to aid in therapeutic education for diabetes patients. Our findings indicate that patients gained statistically significant new knowledge about carbohydrate choices in the short term using our AR application, and it has a suggestively significant impact on mid-term retention. The AR application benefits both children and adults, regardless of gender.

AR applications of this type offer great flexibility in the learning process, as the activity can be conducted anywhere and at any time. Patients only require a minimal setup involving a few printed images and a mobile device. The features of our AR application and its minimal requirements make it a highly accessible educational tool with great potential for therapeutic diabetes education. In this work, we explored the application potential for diabetes therapeutic education. However, there are several avenues for future improvement. Our study assessed participants’ knowledge retention two weeks after the activity. Future research should aim to include a larger sample size and conduct a larger longer-term evaluation, assessing knowledge retention at intervals such as one month, six months, and one year. This would provide deeper insights into the long-term effectiveness of the AR application. Additionally, it should examine how this knowledge acquisition and retention, and consequently carbohydrate counting, affect the patient’s health, for example, by examining glycated hemoglobin (HbA1c) levels. Base on all of those findings, integrating AR applications like ours or other technology-based tools into therapeutic education could be considered, especially to support patients in carbohydrate counting and improve dietary self-management. Furthermore, a larger sample size could support the application of machine learning models to complement the statistical analyses, enabling more advanced insights, such as predictive modeling and pattern recognition in participant behavior and learning outcomes. The application could be improved by adding support for multiple languages and adjusting the carbohydrate choice equivalents based on the user’s country.

## Figures and Tables

**Table 1:** Mann–Whitney U tests for the usability and satisfaction questions between the Ecuadorians and the Spanish patients after using the AR applications.

Variables	Ecuado.	Spanish	*U*	*Z*	*p*	*r*
US#1. I found the AR application easy to use	5; 0.5	4; 2	1078.5	2.909	0.004 *	0.314
US#2. I have gotten used to the application quickly	5; 0	4; 2	1130.5	3.564	0.001 *	0.384
US#3. I have concentrated more on playing than on the Tablet	5; 1	5; 2	901.0	1.088	0.279	0.117
US#4. I could get close enough to the food	5; 1	4; 2	1076.5	2.815	<0.005 *	0.304
US#5. I could see the food from different positions	5; 1	5; 2	967.5	1.786	0.075	0.193
SA#1. I have had a good time	5; 0	5; 1	863.5	0.804	0.425	0.087
SA#2. I liked how the food looked on the dish	5; 0	5; 1	897.0	1.184	0.239	0.128
SA#3. I felt that the food on the dish could be real food	5; 1	5; 1	801.5	0.054	0.961	0.006
SA#4. I think I have learned with this application	5; 0	5; 1	1035.0	2.598	**0.010**	0.280
SA#5. I would like to use these applications to learn more about diabetes	5; 0	5; 1	887.0	1.068	0.288	0.115
SA#6. I would invite my friends to use the application	5; 1	4; 2	1060.5	2.701	**0.007**	0.291
SA#7. I would use this application again	5; 0	5; 1	1030.5	2.498	**0.013**	0.269
SA#8. Score the application from 1 to 5	5; 0	5; 1	972.5	2.077	**0.038**	0.224

Statistically significant results (*p* < 0.005) are shown in bold with the symbol ‘*’. Suggestively significant results (0.005 ≤ *p* < 0.05) are shown in bold. US#1, US#2, etc. refer to Usability questions, while "SA#1, SA#2, etc. refer to Satisfaction questions. The numbers indicate the specific question number within each category.

**Table 2:** Mann–Whitney U tests to assess whether gender affects the scores obtained using the traditional method and the AR application.

	Women	Men	*U*	*Z*	*p*	*r*
Traditional method	4; 2	5; 2	60.0	−1.530	0.132	0.294
AR application	8; 1	8; 1	115.5	1.276	0.211	0.246

**Table 3:** Mann–Whitney U tests assess whether gender affects the UEQ variables.

	Women	Men	*U*	*Z*	*p*	*r*
Pragmatic Quality	2.75; 0.25	3; 0.6875	86.0	−0.260	0.815	0.050
Hedonic Quality	3.00; 0.5	2.625; 0.6875	105.5	0.752	0.468	0.145
Overall	2.875; 0.375	2.75; 0.5	94.0	0.150	0.901	0.029

**Table 4:** Kruskal–Wallis tests for the knowledge scores considering age.

	d.f.	*H*	*p*
Pres-test Traditional	15	15.494	0.416
Post-test Traditional	15	13.357	0.575
Pre-test AR application	15	13.315	0.578
Post-test AR application	15	9.7032	0.838

**Table 5:** Kruskal–Wallis tests for the UEQ variables considering age.

	d.f.	*H*	*p*
Pragmatic Quality	15	12.203	0.664
Hedonic Quality	15	21.842	0.112
Overall	15	18.992	0.214

## Data Availability

The data presented in this study are available upon reasonable request from the corresponding author.
